# A Unique Presentation of Bilateral Sprengel’s Deformity with a Horseshoe Configuration of Connected Omovertebral Bony Bars

**DOI:** 10.3390/jcm15145502

**Published:** 2026-07-14

**Authors:** Abdulmonem Alsiddiky, Hayfaa Saud Alshaalan, Talal Alghadir, Razan Ali Alshatwi

**Affiliations:** 1 Department of Orthopedics, Research Chair of Spinal Deformities, College of Medicine, King Saud University, Riyadh 12372, Saudi Arabia; 2 Department of Orthopedics, College of Medicine, King Saud University, Riyadh 12372, Saudi Arabia; 3 College of Medicine, King Saud University, Riyadh 12372, Saudi Arabia

**Keywords:** Sprengel’s deformity, fused omovertebral bone, Klippel–Feil syndrome, Woodward procedure, bilateral, congenital scapular elevation, horseshoe configuration, pediatric orthopedics

## Abstract

**Background**: Sprengel’s deformity is the most common congenital anomaly of the scapula and is frequently associated with the presence of omovertebral bars and cervical spine anomalies such as Klippel–Feil syndrome. Multiple surgical techniques have been described aiming to correct Sprengel’s deformities, with the Modified Woodward procedure being among the most widely utilized techniques. Bilateral Sprengel’s deformities are relatively rare; when present, surgical correction is usually performed in a staged manner. **Case presentation**: We report a rare case of a three-year-old female with bilateral Sprengel’s deformity associated with Klippel–Feil syndrome, distinguished by a unique horseshoe-shaped fused omovertebral bony bars connecting both scapulae to the cervical spine. The patient underwent simultaneous bilateral Modified Woodward’s procedure with resection of the fused omovertebral bony bars and anchoring both scapulae to the relevant vertebrae. Postoperatively, the patient demonstrated improved shoulder symmetry and full active range of motion at two months of follow-up, with no neurological complications. **Conclusions**: This case reports a previously unreported anatomical variant of bilateral Sprengel’s deformity associated with Klippel–Feil syndrome, characterized by a horseshoe-shaped fused omovertebral bone connecting both scapulae to the cervical spine. Simultaneous bilateral correction using a modified Woodward’s procedure achieved favorable cosmetic and functional outcomes, supporting its feasibility in selected pediatric patients. Thorough preoperative imaging remains critical for identifying complex anatomical variations and optimizing surgical outcomes.

## 1. Introduction

Sprengel’s deformity, also known as congenital elevation of the scapula, is the most common congenital anomaly of the scapula and results from failure of normal caudal migration of the scapula during early embryonic development [1,2]. During fetal development, the scapula originates in the cervical region and normally descends to the upper thoracic region between the ninth and twelfth weeks of gestation [3,4]. When this descent fails to occur, the scapula remains elevated, hypoplastic, and frequently rotated, leading to both cosmetic deformity and functional limitation of shoulder motion [1,2].

Clinically, patients typically present with shoulder asymmetry, restricted shoulder abduction and forward flexion, and prominence of the superomedial scapular angle [5]. The severity of deformity is commonly assessed using the Cavendish classification based on cosmetic appearance [6] and the Rigault classification based on radiographic findings [7]. These classification systems provide standardized methods for evaluating deformity severity, facilitating surgical decision-making and comparison of postoperative outcomes across studies.

Sprengel’s deformity may occur as an isolated condition but is frequently associated with other congenital anomalies, most notably Klippel–Feil syndrome, congenital scoliosis, rib anomalies, and cervical vertebral segmentation defects [8,9]. Klippel–Feil syndrome is characterized by congenital fusion of cervical vertebrae and is among the most frequently reported associated anomalies in patients with Sprengel’s deformity [10]. Both conditions are believed to arise from disturbances in early embryonic development of the paraxial mesoderm, resulting in abnormal vertebral segmentation and impaired scapular descent. This shared embryological origin may explain the frequent coexistence of Sprengel’s deformity and Klippel–Feil syndrome, although the precise developmental mechanisms remain incompletely understood [8,11,12].

An omovertebral connection, consisting of fibrous, cartilaginous, or osseous tissue between the scapula and cervical spine, is present in approximately one-third to one-half of patients [13,14,15]. These abnormal connections tether the scapula to the spine and contribute to the limitation of shoulder abduction and forward flexion [13,14]. Although the exact embryological origin of the omovertebral structure remains controversial, it is generally considered a persistent congenital connection resulting from abnormal scapulovertebral development. Recognition of its presence is clinically important because it contributes to functional limitation and frequently requires excision during surgical correction to achieve adequate scapular mobilization [3,11,13,14].

Surgical management is generally recommended for moderate to severe deformities, particularly in patients with functional limitations or significant cosmetic concerns [16]. Several procedures have been described, including the Green’s procedure [17] and the Woodward’s procedure [18], as well as other modifications designed to reposition the scapula caudally and restore scapulothoracic mechanics [16]. Surgical correction is most commonly performed between three and eight years of age, when soft tissue plasticity allows safer repositioning and better functional outcomes [19,20].

Bilateral Sprengel’s deformity is uncommon, and most reported cases are unilateral [21]. When bilateral deformities are present, correction is typically performed in staged procedures with surgery done on one side followed by the contralateral side several weeks or months later [22,23]. Simultaneous bilateral correction has rarely been reported in the literature. Furthermore, previously reported bilateral cases have described separate unilateral omovertebral connections rather than a single fused osseous structure spanning both scapulae. Consequently, the spectrum of bilateral omovertebral anomalies remains poorly characterized, and additional reports are needed to improve understanding of their anatomical variations, embryological basis, and surgical management [21,22,23].

We present a rare case of bilateral Sprengel’s deformity associated with Klippel–Feil syndrome treated with simultaneous bilateral Modified Woodward’s procedure in a three-year-old child. The case is further distinguished by the presence of bilateral omovertebral bones fused together, forming a horseshoe-shaped structure connecting both scapulae to the cervical spine, an anatomical configuration that, to the best of our knowledge, has not been previously described in the literature.

## 2. Case Presentation

A three-year-old medically free female child was referred to the orthopedic department for evaluation and management of bilateral Sprengel’s deformity. The parents reported noticeable shoulder asymmetry and cosmetic prominence since early childhood, particularly during arm elevation and overhead activities. The child had an unremarkable prenatal, perinatal, and developmental history with no known family history of congenital musculoskeletal disorders. No genetic evaluation was performed.

Physical examination revealed a short neck with bilateral high-riding scapulae with limited abduction and forward flexion of both shoulders (Figure 1). Based on the cosmetic appearance, the deformity was classified as Cavendish Grade III. Right shoulder active abduction was limited to 90° with 100° of forward flexion. Left shoulder active abduction and forward flexion were limited to 110°, without neurovascular compromise. Sensation over the C5 to T1 dermatomes was intact, and motor examination of the shoulder girdle muscles demonstrated no focal weakness.

Anteroposterior X-ray of the spine demonstrated bilaterally elevated, hypoplastic scapulae, consistent with Sprengel’s deformity (Figure 2). According to the Rigault classification, the deformity was classified as Grade III. A computed tomography (CT) scan was performed to provide detailed anatomy, assess the degree of asymmetry, evaluate the presence of omovertebral bars, and facilitate preoperative planning. It revealed bilateral Sprengel’s deformity, more pronounced on the right side, characterized by elevated and axially rotated scapulae. Bilateral fused omovertebral bones were identified at the C3–C4 level, appearing as short, well-corticated bony structures medial to both scapulae, with no bony fusion to the scapulae.

CT also demonstrated multilevel congenital cervical vertebral segmentation anomalies, including rudimentary intervertebral discs at the C2–C3, C3–C4, C4–C5, and mild involvement of the C5–C6 levels. There was osseous fusion of the posterior elements from C2 to C5 on the right side, C2 to C4 on the left side, and the left C5–C6 posterior elements. Non-fusion of the posterior arch of C1 and the lower cervical posterior elements was also observed. These findings established the diagnosis of Klippel–Feil syndrome during the orthopedic evaluation (Figure 3). The remaining visualized upper chest and cervical structures are grossly unremarkable. After multidisciplinary evaluation, the patient was scheduled for simultaneous bilateral Modified Woodward’s procedure.

## 3. Surgical Technique

The patient underwent bilateral Modified Woodward’s procedure under general anesthesia. Considering the patient’s young age, bilateral involvement, and the anticipated benefits of a single anesthetic exposure and a single postoperative rehabilitation period, simultaneous bilateral correction was selected following multidisciplinary evaluation and discussion with the patient’s family regarding the potential risks and benefits of this approach. While placed in a prone position, the surgical field was prepared and draped in a sterile manner, allowing adequate exposure of the posterior neck, back, and bilateral scapulae.

A midline longitudinal posterior incision was performed extending from C1 to T12. Dissection was carried out through the posterior scapular line to access the left scapula. The periscapular muscles were reflected, and the cartilaginous portions of the scapula were resected. Omovertebral bony bar was identified fused with the right side. Then moving on to the right side, the same surgical steps were carried out in a similar manner. Wide posterior exposure of the operative field and identification of the abnormal structures are demonstrated in Figure 4.

The omovertebral bony bars were fused to each other in a horseshoe-shaped configuration and were deeply connected to the spinous process of C3. They were identified from both sides and resected. The resected omovertebral bony bars measured 4.5 × 5.6 cm (Figure 5).

After mobilization of both scapulae, they were caudally repositioned to achieve the desired anatomical alignment. Following the anchoring suture modification described by Alsiddiky et al. [24], both scapulae were secured in their corrected position using No. 2 Vicryl anchoring sutures in each scapula, with a total of 4 Vicryl anchoring sutures. This modification was selected to provide stable fixation of the repositioned scapulae while facilitating controlled rotational alignment during healing. Each suture passed from the inferior medial scapular region, approximately 1 cm lateral to the vertebral border, to the spinous processes of T6–T12 at an angle of 30–40°. Both scapulae were anchored at a point approximating the center of rotation, thereby facilitating both stabilization and rotation. Prior to the final tightening of the sutures, both shoulders were taken through a full range of abduction to ensure appropriate suture tension and to confirm adequate scapular mobility.

## 4. Postoperative Management and Follow-Up

The patient tolerated the procedure well with preserved distal pulses and intact upper limb movements postoperatively. Bilateral body strapping for both upper limbs was applied. Bilateral shoulder immobilization was maintained for 6 weeks postoperatively. Following immobilization, a structured rehabilitation program was initiated, consisting of progressive passive and active range-of-motion exercises under clinical supervision, with gradual return to unrestricted shoulder activities as tolerated. Within the first 24 h after surgery, the patient demonstrated preserved hand grip, finger movements, and elbow flexion and extension bilaterally with no neurological deficit.

At the 2-month follow-up, the patient demonstrated improved shoulder symmetry and full active shoulder range of motion bilaterally, achieving 180° of forward flexion, 180° of abduction, 90° of external rotation, and 70° of internal rotation in both shoulders, without neurological complications. At the most recent follow-up, 6 months postoperatively, the patient maintained full shoulder range of motion, demonstrated satisfactory cosmetic improvement, and remained neurologically intact. The patient’s parents expressed satisfaction with both the cosmetic appearance and functional outcome following surgery. Postoperative anteroposterior radiography demonstrated improved bilateral scapular position and symmetry following simultaneous bilateral correction (Figure 6).

## 5. Discussion

Sprengel’s deformity results from failure of normal scapular descent during early embryologic development, leading to an elevated, rotated, and often dysplastic scapula [1,2]. The resulting deformity may lead to cosmetic asymmetry and restriction of shoulder motion, particularly limitation of abduction [18]. In moderate to severe cases, surgical correction is generally recommended to improve both functional outcomes and shoulder symmetry [16,17]. Early surgical intervention, particularly between three and eight years of age, is generally advocated because of greater soft-tissue elasticity, improved potential for scapular repositioning, and a lower risk of neurovascular complications compared with delayed correction [3,4,5,6,7,8,9,10,11,12,13,14,15,16,17,18,19,20,21,22,23,24,25].

A well-recognized feature of Sprengel’s deformity is the presence of an omovertebral connection, which may consist of fibrous, cartilaginous, or osseous tissue extending from the superomedial border of the scapula to the cervical spine [13,14]. These structures are reported in approximately one-third to one-half of patients [15]. Resection of the omovertebral structure is therefore considered an important step during surgical correction, as it allows adequate mobilization of the scapula and facilitates its caudal repositioning [17,18]. Although the precise embryological origin of the omovertebral structure remains uncertain, it is generally considered a persistent congenital connection resulting from abnormal scapulovertebral development during embryogenesis. Recognition of this structure is clinically important because preoperative identification facilitates surgical planning and complete excision, thereby allowing optimal scapular mobilization and reducing residual tethering that may compromise postoperative correction [3,13,14,15,16].

The anatomical configuration observed in the present case is particularly unusual. Instead of independent unilateral omovertebral bars, the patient demonstrated bilateral omovertebral bones that were fused together, forming a single horseshoe-shaped structure connecting both scapulae to the cervical spine. Bilateral Sprengel’s deformity itself is uncommon, and only a limited number of cases have been reported in the literature. Previously published reports have described bilateral deformities associated with independent bilateral omovertebral bones [21] or bilateral cases managed with simultaneous Modified Woodward procedures without this distinctive anatomical configuration [22]. Other reports have described familial bilateral Sprengel’s deformity without a fused omovertebral structure [23]. To the best of our knowledge, this fused horseshoe-shaped osseous bridge has not been previously described in the literature. Accordingly, the present case broadens the currently recognized spectrum of omovertebral anomalies associated with Sprengel’s deformity and highlights an anatomical variant that surgeons should be aware of during preoperative planning and surgical correction. This fused structure likely contributed to the bilateral restriction of scapular motion observed in this patient and highlights the importance of advanced imaging. CT proved particularly valuable in this case by clearly delineating the complex anatomical relationship between the scapulae and the cervical spine and allowing accurate preoperative planning and safe intraoperative identification of the anomalous osseous connection.

Sprengel’s deformity is also frequently associated with other congenital abnormalities involving the cervical spine and thoracic skeleton. Among the most commonly reported associations is Klippel–Feil syndrome, which is characterized by congenital fusion of cervical vertebrae and segmentation anomalies of the cervical spine [8]. Both conditions are thought to arise from disturbances in early embryonic development of the paraxial mesoderm during the fifth to eighth weeks of gestation, resulting in abnormal vertebral segmentation and impaired caudal migration of the scapula [11,12]. Although the exact embryological mechanism underlying omovertebral bone formation remains incompletely understood, it has been proposed that persistence of an abnormal scapulovertebral connection during embryogenesis may contribute to both the development of the omovertebral structure and restriction of scapular descent [3,4,5,6,7,8,9,10,11]. The presence of a fused horseshoe-shaped omovertebral bridge in our patient may therefore represent an extreme manifestation of this abnormal developmental process, occurring in the setting of concurrent Klippel–Feil syndrome. CT imaging demonstrated multilevel congenital fusion of the cervical vertebrae involving several posterior elements and rudimentary intervertebral disks, consistent with Klippel–Feil syndrome and further emphasizing the complexity of this presentation. These findings are consistent with previously reported associations between Sprengel’s deformity and congenital cervical spine anomalies [26,27].

Although staged correction has traditionally been favored for patients with bilateral Sprengel’s deformity, simultaneous bilateral correction may represent a reasonable alternative in carefully selected patients [22]. Potential advantages include a single anesthetic exposure, avoidance of a second hospitalization and surgical procedure, reduced overall healthcare utilization, and completion of rehabilitation within a single recovery period [16,17,18,19,20,21,22]. However, simultaneous bilateral surgery also requires meticulous preoperative planning, careful intraoperative positioning, and close postoperative monitoring because of the increased operative extent [16,17,18,19,20,21,22]. In the present case, the patient’s young age, good general health, and absence of significant medical comorbidities supported the decision to proceed with simultaneous bilateral correction. The favorable functional and cosmetic outcomes observed at the 6-month follow-up further support the feasibility of this approach in appropriately selected patients, although larger studies with longer follow-up are needed before broader recommendations can be made [3,19,20].

Among the various surgical techniques described for the correction of Sprengel’s deformity, Woodward’s procedure remains one of the most widely used [17,18]. The procedure involves detachment and inferior transfer of the trapezius and rhomboid muscle origins from the spinous processes, allowing the scapula to be repositioned caudally and improving scapulothoracic mechanics [25]. Several modifications have been proposed to enhance fixation stability and optimize postoperative outcomes [24,25,26,27,28].

In the present case, the anchoring suture modification described by Alsiddiky et al. [24] was used to stabilize the repositioned scapulae. This technique utilizes anchoring sutures between the medial scapular border and thoracic spinous processes to maintain the corrected position while allowing controlled rotational alignment of the scapula. The favorable intraoperative findings and absence of postoperative neurological complications in this patient support the safety and effectiveness of this modification.

This case is notable for several distinctive features. First, bilateral Sprengel’s deformity remains relatively uncommon [21,26,27]. Second, the presence of bilateral omovertebral bones fused together into a horseshoe-shaped structure connecting both scapulae to the cervical spine represents an unusual anatomical variant that, to the best of our knowledge, has not been previously described in the literature. This unique anatomical configuration expands the currently recognized spectrum of omovertebral anomalies associated with Sprengel’s deformity and underscores the importance of meticulous preoperative imaging in identifying complex anatomical variations that may influence surgical planning. Finally, the favorable cosmetic and functional outcomes observed at the 6-month follow-up suggest that simultaneous bilateral Modified Woodward procedures represent a feasible surgical option in carefully selected pediatric patients.

Several limitations should be acknowledged. As a single case report, the findings cannot be generalized to all patients with bilateral Sprengel’s deformity. Although the patient demonstrated excellent cosmetic and functional outcomes at the 6-month follow-up, longer-term surveillance is necessary to evaluate the durability of the surgical correction, maintenance of shoulder function, and skeletal growth. Furthermore, genetic evaluation was not performed, precluding further assessment of potential underlying syndromic or genetic contributors to this rare presentation. Nevertheless, the unique anatomical findings and successful surgical management presented in this case provide valuable insights that may assist surgeons in recognizing and managing similar complex presentations.

Overall, this report expands the spectrum of anatomical variations associated with Sprengel’s deformity and suggests that simultaneous bilateral correction using a Modified Woodward’s technique may be a feasible treatment option in carefully selected pediatric patients. In addition, it highlights the importance of comprehensive preoperative imaging in identifying complex anatomical variations and facilitating individualized surgical planning.

## 6. Conclusions

This case describes a rare anatomical variant of bilateral Sprengel’s deformity associated with Klippel–Feil syndrome, characterized by a horseshoe-shaped fused omovertebral bone connecting both scapulae to the cervical spine. To the best of our knowledge, this anatomical configuration has not been previously reported in the published literature. Successful simultaneous bilateral correction using a Modified Woodward’s procedure resulted in favorable short-term cosmetic and functional outcomes and suggests that single-stage surgery may be a safe and effective option in carefully selected pediatric patients. Careful preoperative imaging is essential for identifying complex anatomical variations and facilitating appropriate surgical planning.

## Figures and Tables

**Figure 1:**
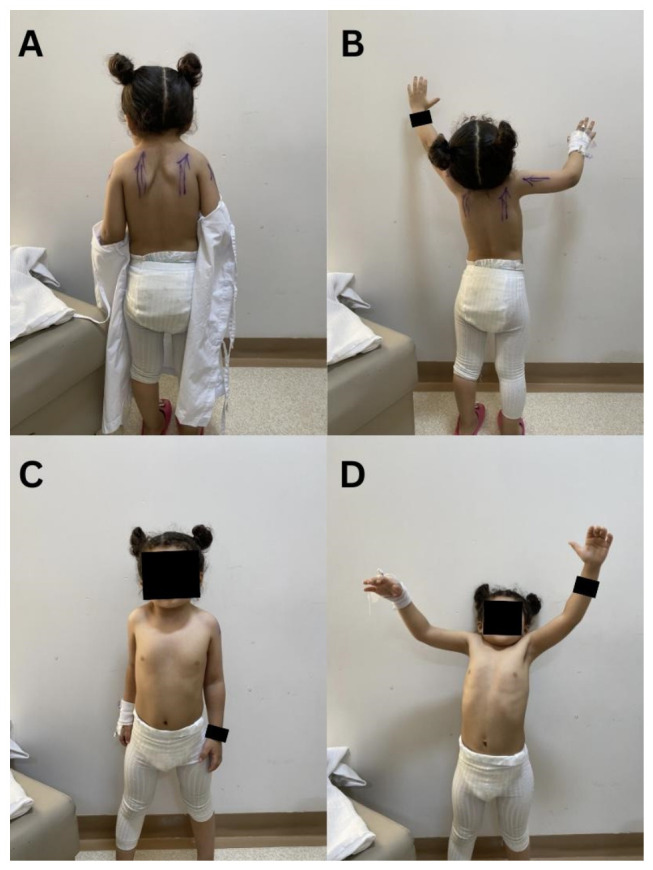
Preoperative clinical appearance of the patient. (**A**) Posterior view demonstrating bilateral elevation of the scapulae. (**B**) Posterior view during attempted shoulder abduction showing restricted elevation of the shoulders. (**C**) Anterior view demonstrating shoulder asymmetry. (**D**) Anterior view during attempted arm elevation demonstrating limitation of overhead shoulder abduction.

**Figure 2:**
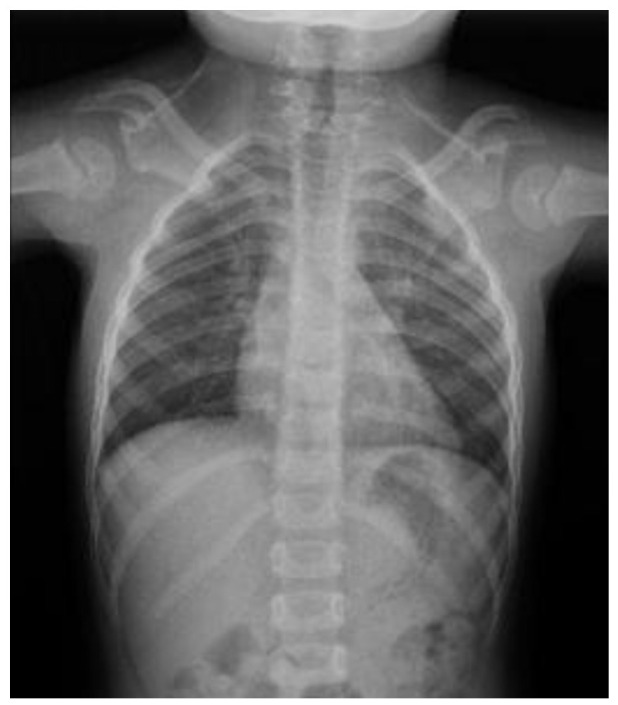
Preoperative anteroposterior X-ray demonstrating bilaterally elevated hypoplastic scapulae consistent with Sprengel’s deformity.

**Figure 3:**
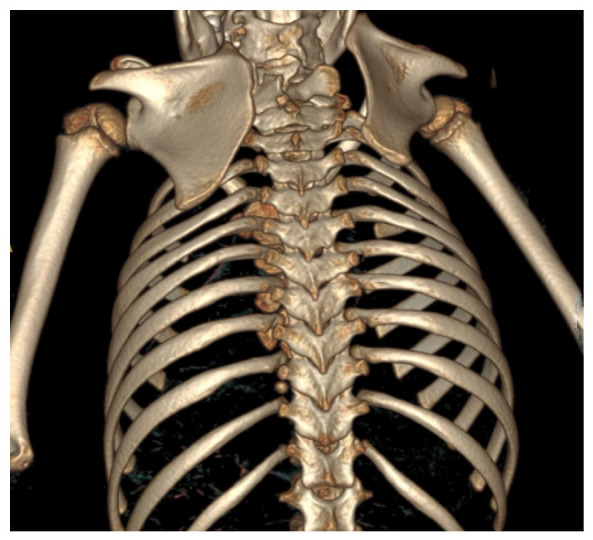
Preoperative computed tomography with three-dimensional reconstruction demonstrating bilateral elevated, axially rotated hypoplastic scapulae, bilateral fused omovertebral bones, and associated cervical vertebral segmentation anomalies consistent with Klippel–Feil syndrome.

**Figure 4:**
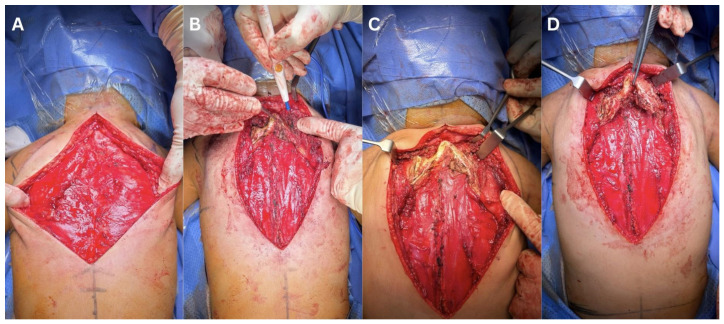
Intraoperative exposure during bilateral Modified Woodward procedure. (**A**) Posterior midline exposure after elevation of the skin flaps and paraspinal musculature. (**B**) Careful dissection using electrocautery to identify the anomalous omovertebral structures. (**C**) Deeper dissection demonstrating exposure of the abnormal bony connection. (**D**) Complete operative exposure prior to resection of the fused omovertebral bars.

**Figure 5:**
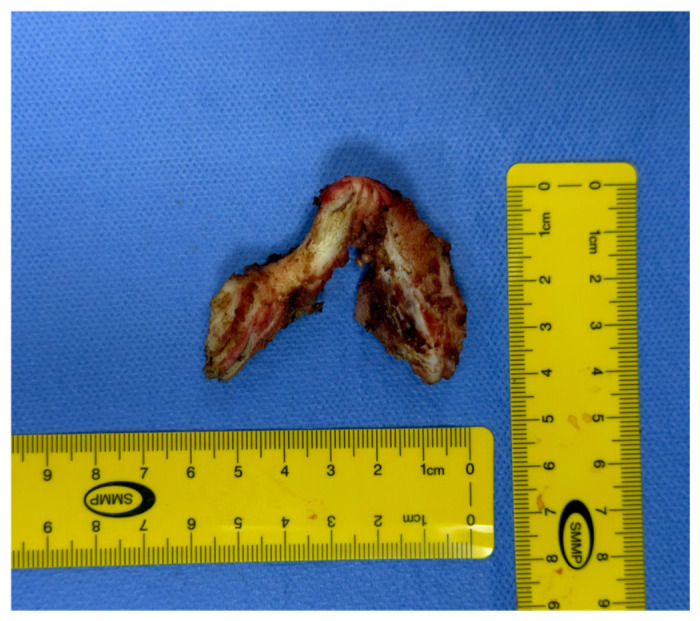
Resected fused omovertebral bone measuring approximately 4.5 × 5.6 cm, demonstrating the characteristic horseshoe-shaped configuration connecting both scapulae.

**Figure 6:**
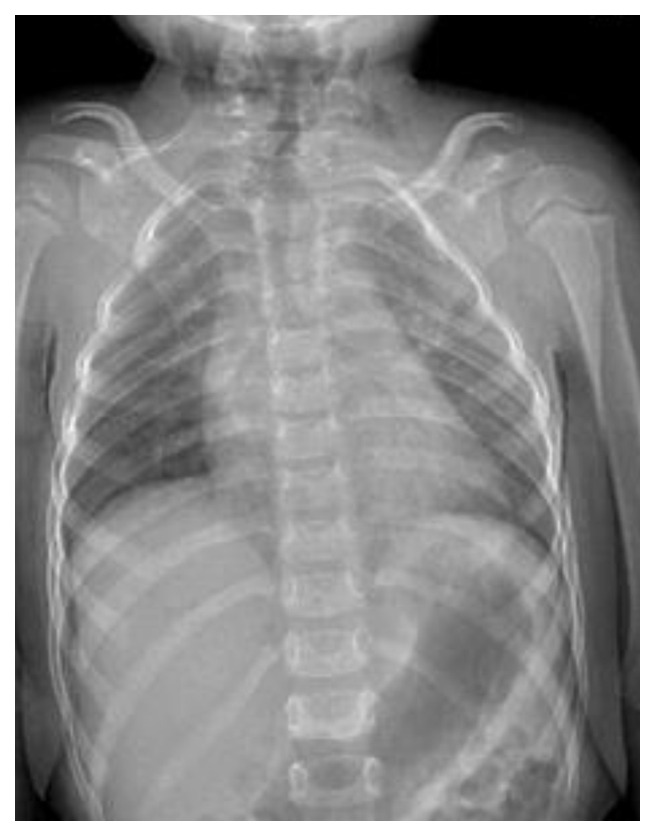
Postoperative anteroposterior radiograph demonstrating improved bilateral scapular position and symmetry following simultaneous bilateral Modified Woodward’s procedure.

## Data Availability

All data generated or analyzed during this study are included in this published article. Further details are available from the corresponding author upon reasonable request, subject to ethical and privacy considerations.

## Abbreviation

The following abbreviation is used in this manuscript:

CT	Computed Tomography
